# Efficacy and safety of cross-linked acellular porcine corneal stroma for infectious corneal disease in China: a multicenter prospective clinical trial

**DOI:** 10.1097/JS9.0000000000003082

**Published:** 2025-07-17

**Authors:** Yunxiao Zang, Lei Tian, Liya Wang, Yueqin Zhang, Lulu Wang, Ting Huang, Xiaojuan Dong, Jing Wu, Chaoran Zhang, Jianjiang Xu, Xipeng Guan, Xingxia Zhong, Lei Zhang, Ying Jie

**Affiliations:** a Beijing Institute of Ophthalmology, Beijing Tongren Eye Center, Beijing Tongren Hospital, Capital Medical University and Beijing Ophthalmology & Visual Sciences Key Laboratory, Beijing, China; b Department of Ophthalmology, Henan Eye Hospital, Henan Provincial People’s Hospital, Zhengzhou, China; c State Key Laboratory of Ophthalmology, Clinical Research Center, Zhongshan Ophthalmic Center, Sun Yat-Sen University, Guangzhou, China; d Department of Otolaryngology-Head and Neck Surgery, Eye Ear Nose and Throat Hospital, SFudan University, Shanghai, China; e Ushine Regenerative Medical Technologies Co., Ltd., Guangzhou, China

**Keywords:** cellular porcine corneal stroma, China, cohort study, efficacy and safety, infectious corneal disease, ophthalmology

## Abstract

**Background:**

Acellular porcine corneal stroma (APCS) is a promising alternative to allografts; however, its potential requires verification. To evaluate the efficacy and safety of novel cross-linked APCS transplantation for the treatment of infectious corneal disease.

**Methods:**

This prospective, multicenter, single-arm clinical trial was conducted from 2015-2019. Ninety patients with infectious corneal disease who had undergone anterior lamellar transplantation with an APCS across four centers in China were enrolled. All patients had previously received ineffective drug treatment and had a preoperative best-corrected visual acuity (BCVA) < 20/400. The primary outcome was the deblinding rate at 180 days compared to the target rate of 65%. The secondary outcomes included BCVA, corneal transparency, and epithelial healing. Safety endpoints included corneal graft rejection, blood count, liver and kidney function, immunoglobulin levels, concomitant medications, and additional treatments. Follow-up was performed for at least 6 months postoperatively.

**Results:**

A total of 100% (90/90) and 88.9% (80/90) of the cases were included in the safety analysis and efficacy per protocol sets, respectively. Eighty percent (72/90) of patients completed 180 days of follow-up after APCS transplantation. The deblinding rate at 180 days was 90.3% (95% confidence interval 81–96%), exceeding the target (*P* < 0.001). At 180 days, 55% of the grafts were completely transparent and 82.5% of the epithelium had no defects. The log of the minimum angle of resolution vision improved from 2.19 ± 0.25 preoperatively to 1.10 ± 0.3. No serious adverse events occurred.

**Conclusion:**

APCS demonstrated excellent corneal transparency and epithelialization following keratoplasty, confirming its safety and efficacy. Six-month data suggest its potential as an alternative treatment for infectious corneal disease. Further long-term trials are necessary to establish its comparative effectiveness and evaluate chronic complications, offering valuable insights for future clinical practice.

## Introduction

Infectious corneal disease is one of the five leading causes of visual impairment worldwide[1]. In China, approximately 85% of corneal blindness cases originate from infectious corneal diseases, with an annual increase of 100 000 patients (The Second China National Sample Survey on Disability, China Association of Persons with Visual Disabilities, 2006). Infectious transplantation is the most effective treatment for patients with corneal blindness[2]. However, allogeneic corneal grafts are scarce in clinical practice[3]. Therefore, many patients with infectious corneal diseases cannot receive timely treatment.

Over the years, researchers have actively explored alternatives to allogeneic corneal grafts for treating corneal diseases. Keratoprostheses (Boston Type I and II) and acellular porcine corneal stroma (APCS) have emerged as two of the most promising products^[4,5]^. Clinical trial data show that most patients regain their vision after implantation[6]; however, the incidence of postoperative complications is closely related to the surgical indications of the primary infectious corneal disease[7]. In comparison, APCS has numerous advantages including a more abundant source, better biocompatibility, stronger regenerative capability, fewer postoperative complications, and lower cost[4].

The first clinical trial applying APCS for infectious corneal diseases in 2015 demonstrated its application potential. Nevertheless, the trial found that the optical transparency and mechanical strength required improvements[8]. In recent years, advances in decellularization[9] and crosslinking techniques[10] have improved the transparency and mechanical stability of APCS products[11]. However, the clinical indications and safety of APCS transplantation require further clarification via standardized clinical trials[12]. Therefore, herein we aimed to evaluate the efficacy and safety of APCS transplantation for treating mild to moderate infectious corneal diseases, with a focus on the deblinding rate and postoperative clinical characteristics. This was the largest multicenter clinical trial to date, ensuring improved validity.

## Methods

### Ethics statement

The clinical study protocol and amendments (Supplemental Digital Content, Supplement 1, available at: http://links.lww.com/JS9/E716) were reviewed and approved by independent ethics committees and institutional review boards at each site. The study followed the guidelines of the International Council for Harmonization Good Clinical Practice and the Declaration of Helsinki. Written informed consent was obtained from each participant before enrollment in the study.

### Study design and participants

We conducted a prospective, multicenter, open-label clinical trial to determine the efficacy and safety of APCS transplantation for the treatment of infectious corneal diseases. This study was retrospectively registered after its initiation (ClinicalTrials.gov Identifier: ***). Despite the delayed registration, the entire research process strictly adhered to the original study protocol, with no deviations. In this study, APCS, classified as a Class I medical device, has been registered with the Center for Medical Device Evaluation (NMPA) of the National Medical Products Administration (NMPA) in China and has obtained approval, including the complete clinical trial report (*** Food and Drug Administration: 20223160613). The details of the clinical protocol are provided in Supplemental Digital Content, Supplement 1, available at: http://links.lww.com/JS9/E716. This work has been reported in line with the Strengthening the Reporting of Cohort Studies in Surgery (STROCSS) criteria[13]. In this study, 90 patients with infectious keratitis involving the partial thickness of the cornea were enrolled from 2015 to 2019 at four centers in China. Patients were eligible if they had an uncontrolled corneal infection after non-surgical treatment for >2 weeks. The more severely affected eye was used as the study eye. This study was designed as a superiority trial, with the primary objective to demonstrate that APCS transplantation achieves a deblinding rate greater than 65%^[14,15]^, surpassing a predefined efficacy threshold. This approach is consistent with the goal of establishing the intervention’s superiority over a historical benchmark or threshold, as explained in Supplemental Digital Content, Supplement 2, available at: http://links.lww.com/JS9/E717. Patients were enrolled consecutively until a predetermined sample size was reached, which was calculated to provide 80% power to detect a 65% deblinding rate after APCS transplantation at 5% significance level. The rationale for using 65% and 80% can be found in Supplemental Digital Content, Supplement 2, available at: http://links.lww.com/JS9/E717.

**HIGHLIGHTS**

- Approach for infectious corneal diseases: This study demonstrates the efficacy and safety of cross-linked acellular porcine corneal stroma (APCS) transplantation for treating infectious corneal diseases.
- High deblinding success rate: APCS transplantation achieved a deblinding rate of 90.3% at 180 days, significantly exceeding the target rate of 65%.
- Enhanced visual outcomes: The mean BCVA improved significantly from 2.19 logMAR preoperatively to 1.10 logMAR at 180 days postoperatively.
- Optimized biocompatibility and healing: APCS grafts showed excellent corneal transparency, epithelial healing (82.5%), and low rates of graft melting (3.3%).
- Safe and effective alternative: APCS transplantation was associated with minimal complications and could serve as a viable substitute for allogeneic corneal grafts in selected cases.

The inclusion criteria for clinical trial patients were as follows: (1) preoperative best-corrected visual acuity (BCVA) < 20/400 (Snellen), corneal lesions located in the pupillary zone; (2) active infectious corneal disease (fungal, bacterial, viral, and Acanthamoeba keratitis) involving the corneal stroma but not the full thickness; (3) aged 18–80 years old; and (4) uncontrolled corneal infection after non-surgical treatment for more than 2 weeks. The key exclusion criteria were severe corneal vascularization, severe dry eye, concurrent infectious endophthalmitis, and other criteria outlined in the clinical study protocol (Supplemental Digital Content, Supplement 1, available at: http://links.lww.com/JS9/E716).

### Procedures

The APCS used in this study was obtained from porcine corneas. The preparation involved viral inactivation, decellularization, and chemical cross-linking according to the standard operating procedures in an ISO Class 5 cleanroom. The of APCS products were ANCO-250-10, ANCO-400-10, and ANCO-500-10 (representing APCS thicknesses of 250 ± 100 μm, 400 ± 100 μm, and 500 ± 100 μm, respectively). APCS was sealed in a sterile plastic container with antimicrobial solution, sterilized, and stored at −20°C. The storage period before use was approximately 3–10 months. Preoperative ulcer assessment was performed using anterior segment optical coherence tomography (AS-OCT) to guide APCS graft sizing. All surgeries followed a standardized protocol, including AS-OCT-based graft selection and uniform suturing techniques. Procedures were conducted by senior corneal specialists at each site, with all surgeons receiving centralized pre-trial training to ensure technical consistency across centers. After hydration, corneal grafts were harvested using Hessburg–Barron vacuum trephines with adjustable cutting depths. The graft diameter used was 1–2 mm larger than the edema zone to ensure complete removal of the lesion. The grafts were positioned on the epithelial side in the recipient beds and secured with 12-16 interrupted 10-0 nylon sutures. The timing of suture removal was determined based on each patient’s wound healing progression.

Post-transplant medications: nonviral keratitis – systemic antibiotics for 1–2 weeks based on sensitivities, topical antibiotics for ≥1 month. Herpetic keratitis – oral and topical antivirals (acyclovir) for ≥3 months. Fungal keratitis: withhold topical corticosteroids for 2 weeks; otherwise, start topical prednisolone and tacrolimus immediately for 6–12 months.

Patients who underwent APCS transplantation were monitored preoperatively and follow-up evaluations were conducted 3, 7, 30, 60, 90, and 180 days after surgery. The minimum postoperative follow-up period was 6 months, with the final follow-up completed on 19 May 2020 (Supplemental Digital Content, eTable 1, available at: http://links.lww.com/JS9/E721).

### Outcomes

The primary efficacy endpoint was the deblinding rate at 180 days post-operation, defined strictly as the proportion of cases with BCVA ≥20/400 out of the total cases with visual acuity data. This was compared to the target deblinding rate of 65%. The primary secondary efficacy and safety endpoints included best-corrected log of the minimum angle of resolution (logMAR) vision, control of the primary infection, corneal clarity, edema, neovascularization, corneal epithelial healing, conjunctival hyperemia, intraocular pressure (IOP), and ocular irritation symptoms at each follow-up. The grading scales for these indices are detailed in Supplemental Digital Content, Supplement 1, available at: http://links.lww.com/JS9/E716. Other safety endpoints included corneal graft rejection, blood count, liver and kidney function, immunoglobulin levels, concomitant medications, and additional treatment. Detailed safety endpoints are described in Supplemental Digital Content, Supplement 1, available at: http://links.lww.com/JS9/E716.

### Statistical analysis

The binary primary endpoint was analyzed by comparing it to a hypothetical success rate of 80% using a one-sided binomial test at a significance level of 0.025 (Supplemental Digital Content, Supplement 2, available at: http://links.lww.com/JS9/E717). With 80% power, a sample size of at least 72 subjects was required based on calculations in nQuery Advisor + nTerim 3.0 (Statistical Solutions, USA). Accounting for up to 20% dropouts, the target sample size was set to 90 subjects.

All analyses were conducted using SAS software (version 9.4, SAS Institute). Continuous variables are summarized as mean ± standard deviation and categorical variables as frequency and percentage. The primary efficacy endpoint of the deblinding rate at 180 days was analyzed using a one-sided binomial test with a hypothetical success rate of 80%. The secondary efficacy and safety endpoints were subjected to descriptive statistical analysis. Pre-specified subgroup analyses stratified outcomes by study center, product models and specifications, and infection type. Sensitivity analyses were performed on the per-protocol population (n = 72), using two approaches to handle missing data. One-way analysis was performed using Pearson chi-square tests. All other statistical tests (except primary endpoint) were two-sided, and significance was defined as *P* <0.05.

## Results

### APCS characteristics and patients

The cross-linked APCS replacements exhibited excellent tensile strength, stability, and low degradation, ensuring integrity during surgical use. Chemical properties, including pH and heavy metal content, were within acceptable limits. Biological testing revealed no significant endotoxin levels, cytotoxicity, or sensitization, indicating good biocompatibility. Animal studies with rabbit corneal defects demonstrated successful epithelialization and restoration of transparency post-APCS transplantation. These results confirm the safety and efficacy of APCS for corneal repair applications (Supplemental Digital Content, Supplement 3, available at: http://links.lww.com/JS9/E718 and Supplemental Digital Content, eFigure 1, available at: http://links.lww.com/JS9/E720).

**Figure 1.**
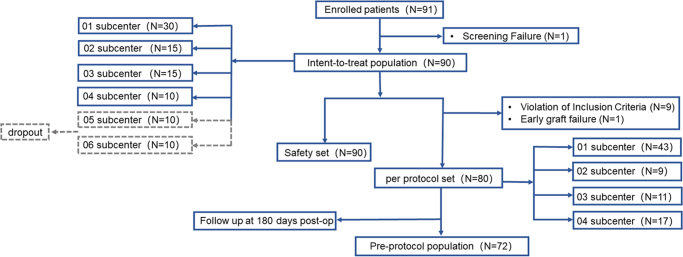
Flow diagram of study population inclusion.

The screening and enrollment flowchart is shown in Figure 1. Ninety-one participants were enrolled in this study and nighty patients were included in the safety set for safety assessment of APCS transplantation. After excluding 10 participants who met the exclusion criteria and 1 participant who had early graft failure leading to study discontinuation, 80 participants (88.9%) who received APCS transplantation were included in the modified intent-to-treat population (MITTP) for statistical analysis. Of the 7 patients who did not complete the 180-day follow-up, 5 (71.4%) missed visits due to COVID-19 restrictions, 1 (14.3%) required conjunctival flap coverage for recurrent fungal infection, and 1 (14.3%) needed conjunctival flap coverage due to APCS graft rejection and dissolution (included in the safety analysis). A total of 72 participants (80%) successfully completed the 180-day follow-up after APCS transplantation.

01, indicates Henan eye hospital; 02, Beijing Tongren hospital; 03, Zhongshan Ophthalmic Center; 04, Eye and ENT Hospital of Fudan University; 05, The Southwest hospital; 06, Tianjin Medical University Eye Hospital. ENT, ear-nose-throat.

The mean age of the 80 participants was 59 ± 9 years. The percentage of males and females was 58.8% (47/80) and 41.3% (33/80), respectively. All 80 patients had uncontrolled primary corneal infections (2 cases of bacteria, 53 cases of fungi, 2 cases of virus, and 23 cases of unknown etiology). Eleven patients (13.8 %) had moderate corneal opacity, and 68 patients (85%) had severe opacity. The mean diameter of the corneal lesion was 6.82 ± 0.98 mm. The mean BCVA in logMAR units was 2.19 ± 0.25, indicating poor visual acuity before treatment (The conversion between Snellen and logMAR visual acuity can be found in Supplemental Digital Content, Supplement 1, available at: http://links.lww.com/JS9/E716).

The mean graft bed size was 6.87 ± 0.93 mm and the mean graft size was 7.04 ± 0.95 mm. Ocular comorbidities included none (42,52.5%), cataract (35,43.8%), glaucoma (1,1.3%), strabismus (1,1.3%) and corneal dystrophy (1,1.3%), respectively. None of the patients had hypopyon. In one patient with glaucoma, intraocular pressure was well controlled with two types of topical antiglaucoma medications. The surgical success rate was 100% (80/80 patients). The ANCO-250-10, ANCO-400-10, and ANCO-500-10 product specifications were used in 7.5% (6/80), 82.5% (66/80), and 10.0% (8/80) of the cases, respectively. Detailed baseline characteristics and APCS transplantation data are presented in Tables 1 and 2, respectively.

**Table 1:** Baseline demographic and disease characteristic (MITTP, n = 80)

Characteristic	No. (%) Participants group
Age, mean ± SD, y	59 ± 9
Gender	
Male	47 (58.8)
Female	33 (41.3)
Occupation	
Manual laborer	67 (83.8)
Non-manual laborer	13 (16.3)
Lesion location^a^	
Left eye	46 (57.5)
Right eye	34 (42.5)
Lesion size (mm)	
Diameter, mean ± SD (range)	6.82 ± 0.98 (2.0-9.0)
BCVA (logMAR), mean ± SD	2.19 ± 0.25
Visual acuity grading(Snellen VA)	
Blindness (BCVA<20/400)	80(100)
Primary corneal infection	
Bacterial	2(2.5)
Fungal	53(66.3)
Viral	2(2.5)
Other (undetermined pathogen) b	23(28.8)
Control of primary corneal infection	
Uncontrolled	80(100)
Corneal clarity c	
Grade 0 (transparent)	0(0)
Grade 1 (mild opacity)	1(1.3)
Grade 2 (moderate opacity)	11(13.8)
Grade 3 (severe opacification)	68(85)
Ocular irritation symptoms	
Grade 0 (no irritation)	0(0)
Grade 1 (mild irritation)	2(2.5%)
Grade 2 (moderate irritation)	13(16.3%)
Grade 3 (severe irritation)	65(81.3%)
Ocular comorbidities	
None	42(52.5)
Cataract	35(43.8)
Glaucoma	1(1.3)
Strabismus	1(1.3)
Corneal dystrophy	1(1.3)
History of corneal transplantation	
None	78(97.5)
Yes (ALK)	2(2.5)
History of allergy	
No	76(95)
Yes	4(5)

ALK = anterior lamellar keratoplasty; BCVA = best-corrected visual acuity; LP = light perception; MITTP = modified intent-to-treat population; NP = no perception; SD = standard deviation; y = years.

^a^ The eye with more severe condition was selected as the study eye.

^b^ Other refers to patients with undetermined pathogen and ineffective empirical treatment for 2 weeks.

^c^ Grading criteria for corneal clarity and ocular irritation symptoms are described in Supplemental Digital Content, Supplementary Methods 1, available at: http://links.lww.com/JS9/E716.

**Table 2:** Characteristics of APCS transplantation (MITTP, n = 80)

Characteristic	No. (%) Participant group
Type of anesthesia	
Local	59 (73.8)
General	21 (26.3)
Recipient bed (mm) mean ± SD (range)	6.87 ± 0.93 (3.25-8.75)
Graft size (mm) mean ± SD (range)	7.04 ± 0.95 (3.5-9.0)
APCS type n (%)^a^	
ANCO-250-10	6 (7.5)
ANCO-400-10	66 (82.5)
ANCO-500-10	8 S(10)
Surgery success	80(100)

APCS = acellular porcine corneal stroma; MITTP = modified intent-to-treat population; SD = standard deviation.

^a^ APCS types, ANCO-250-10, ANCO-400-10, ANCO-500-10 represent decellularized corneas with thicknesses of 250 ± 100 μm, 400 ± 100 μm, and 500 ± 100 μm, respectively. Different types of corneal grafts were chosen during surgery based on the depth of the corneal lesion.

### Primary endpoints

**Figure 2.**
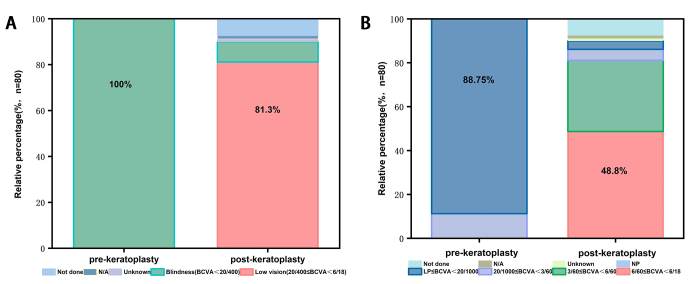
Visual acuity characteristics before and after APCS transplantation.

The deblinding rate at 180 days post-APCS transplantation was 90.3% (95% CI:81 · 0%, 96 · 0%), which was significantly higher than the target deblinding rate of 65% (*P* < 0.001). The mean BCVA in logMAR units was 1.10 ± 0.36 at 180 days post-transplantation in the MITTP. The distribution of BCVA grading was as follows: grade 1 (6/60 ≤ BCVA<6/18), 48.8%; grade 2 (3/60 ≤ BCVA<6/60), 32.5%; grade 3 (20/1000 ≤ BCVA<3/60), 5.0%; grade 4 (LP≤BCVA<20/1000), 3.8%; and grade 5 (NP), 0%. Low vision was achieved in 81.3% (65/80) of participants, whereas 8.8% (7/80) were blind at 180 days (Fig. 2).

Visual acuity grading before and after keratoplasty (A). Visual acuity classification before and after keratoplasty (B). APCS, acellular porcine corneal stroma; BCVA, best-corrected visual acuity; LP, light perception; NP, no perception; N/A, not applicable.

The deblinding rate at 12 months and 24 months post-APCS transplantation were 83.3% (95% CI:70.7 %, 92.1%) and 92.9% [95% CI: 66.1%, 99.8%] respectively, which were all significantly higher than the target deblinding rate of 65% (*P* < 0.001). This section is presented in Supplemental Digital Content, Supplement 1, available at: http://links.lww.com/JS9/E719.

### Secondary endpoints

**Figure 3.**
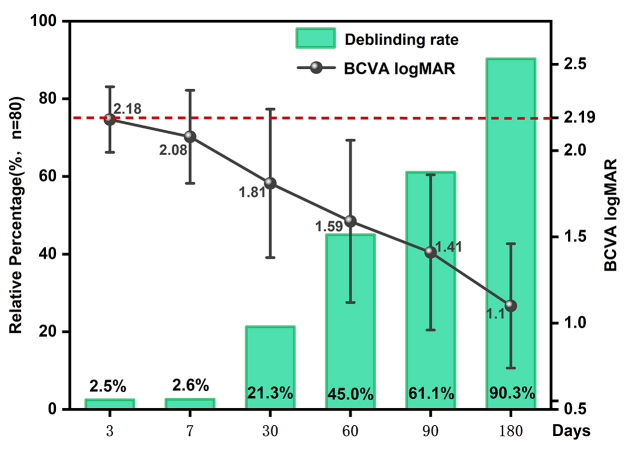
Visual acuity characteristics at different follow-up times after APCS transplantation.

**Figure 4.**
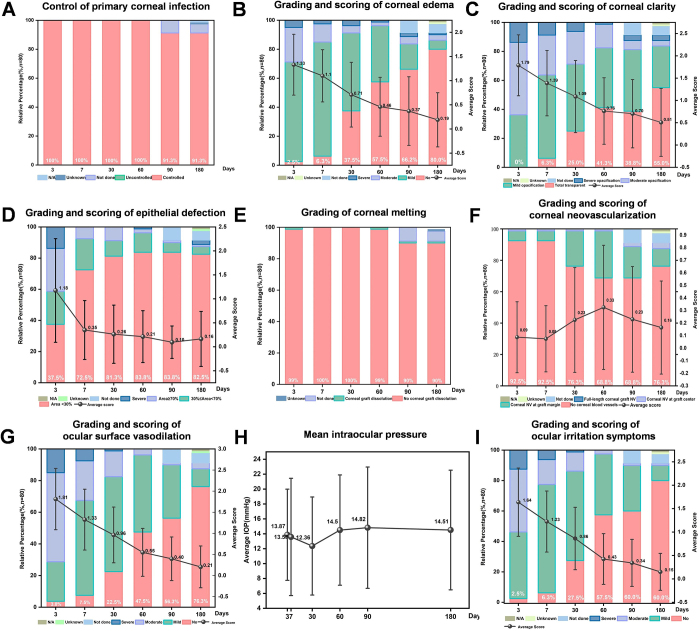
Clinical characteristics at different follow-up times after APCS transplantation.

**Figure 5.**
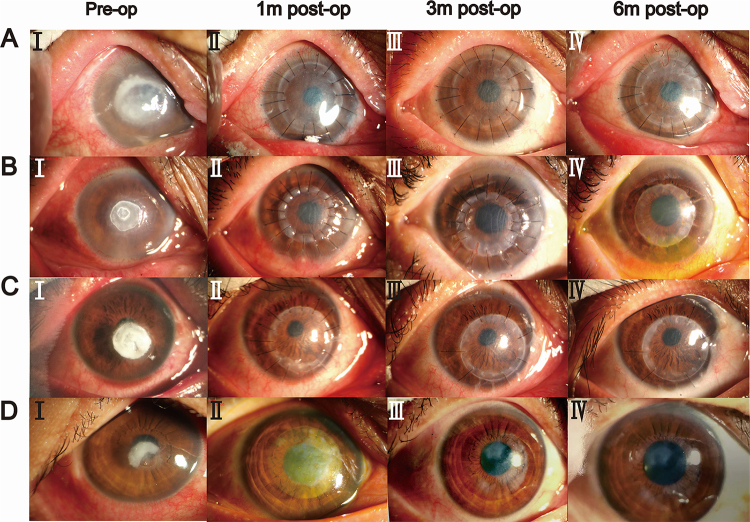
Graft clarity at different follow-up times after APCS transplantation from four different patients.

**Figure 6.**
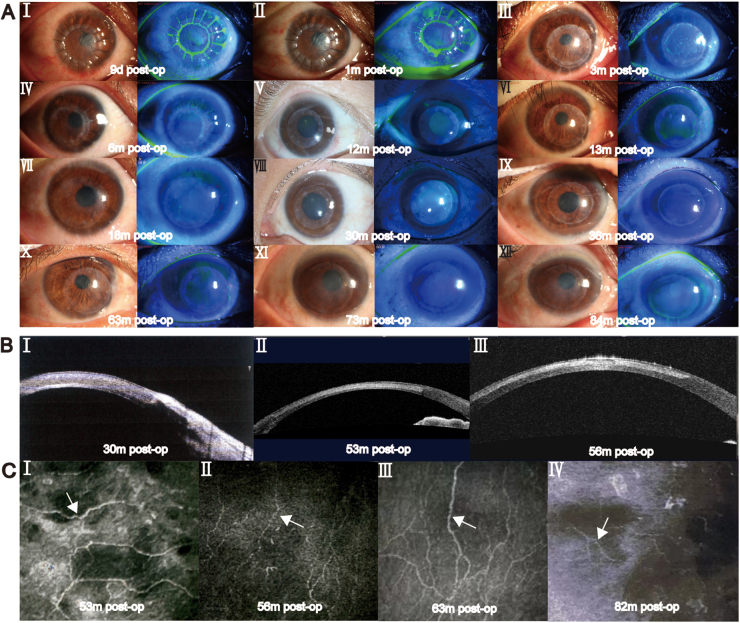
Epithelial integrity at different follow-up times after APCS transplantation from different patient.

At 180 days after APCS transplantation, 96.3% of the patients had improvements in BCVA compared with preoperative values. The deblinding rate, mean BCVA (logMAR), and BCVA at each follow-up are shown in Figure 3. Complete corneal clarity was achieved in 44 (55.0%) patients. An intact corneal epithelium was observed in 66 patients (82.5%). The incidences of neovascularization, corneal edema, ocular surface vasodilation, ocular surface irritation, and corneal melting at each follow-up are shown in Figure 4. The mean IOP after post-keratoplasty at 180 days postoperatively was 14.5 ± 8.0 mmHg. The representative slit-lamp photographs are shown in Figure 5. The results of APCS transplantation with follow-up of 12 months or more are presented in Supplemental Digital Content, Supplemental 4, available at: http://links.lww.com/JS9/E719. Figure 6 shows the graft transparency and epithelial integrity at different follow-up times after APCS transplantation from different patients.

Mean BCVA, logMAR visual acuity, and deblinding rates at different follow-up time points after keratoplasty. Red line: mean preoperative BCVA, logMAR visual acuity. APCS, acellular porcine corneal stroma; BCVA, best-corrected visual acuity; logMAR, log of the minimum angle of resolution.

The grading criteria for corneal edema, corneal clarity, epithelial defects, neovascularization, conjunctival congestion, and ocular irritation are described in Supplemental Digital Content, Supplemental File 1, available at: http://links.Lww.Com/JS9/E716. additional clinical study protocol. Control of primary corneal infection (A). Grading and scoring of corneal edema (B). Grading and scoring of corneal clarity (C). Grading and scoring of epithelial defection (D). Grading of corneal melting (E). Grading and scoring of corneal neovascularization (F). Grading and scoring of ocular surface vasodilation (G). Mean intraocular pressure (H). Grading and scoring of ocular irritation symptoms (I). APCS, acellular porcine corneal stroma; N/A, not applicable.

Preoperative corneal lesion was located in central pupil area, significantly affecting vision (AI, BI, CI, and DI). Sutures were still in place at 3 months after transplantation, indicating sufficient tensile strength of APCS (AIII, BIII, CIII, and DIII). The graft had adequate early clarity after keratoplasty (AIV, BIV, CIV and DIV). APCS, acellular porcine corneal stroma; m, months.

Slit-lamp photographs showing epithelial healing and graft clarity over time. Graft clarity improved progressively and became comparable to that of the surrounding recipient cornea over long-term follow-up (A). Anterior segment optical coherence tomography (AS-OCT) showing well-attached grafts with normal thickness and no anterior chamber inflammation 12 months post-transplantation (B). In vivo confocal microscopy images showing nerve fiber ingrowth (arrows) in the APCS graft observed 24 months post-transplantation (C). APCS, acellular porcine corneal stroma; d, days; m, months.

### Adverse events

During180 days follow-up, three (3.3%) adverse events related to the trial occurred, including two cases of graft rejection and one case of corneal melting. With appropriate intervention, two out of the three cases fully recovered. The third patient did not respond to treatment and underwent a second allogeneic corneal transplantation because of their worsening condition (Table 3). A summary of all adverse events is provided in Supplemental Digital Content, eTable 2, available at: http://links.lww.com/JS9/E722. There were no abnormalities in laboratory parameters (blood cell counts, liver and kidney function tests, and immunoglobulin levels) before or after APCS transplantation. The safety parameters are summarized in Supplemental Digital Content, eTable 3, available at: http://links.lww.com/JS9/E724.

**Table 3:** Occurrences of 3 adverse events related to the trial (safety set n = 90)

Central No.	Gender	Age	Type of infection	Recipient bed/Graft size	Product thickness	Adverse events	Duration	Therapeutic measure	Clinical features (180 days post-op)	Outcome of adverse events	Possible relationship with trial^a^	Whether it meets the criteria for serious adverse event
01-011	Female	60	Fungal	7.25/7.0	ANCO-400-10	Transplant rejection; corneal melting	15 days	Medication ineffective, bio-corneal graft removal and autologous conjunctival flap transplantation	N/A	Worsened	Possibly related	Yes
01-037	Male	50	Fungal	6.75/6.5	ANCO-400-10	Corneal melting	20 days	Medication treatment, wearing corneal contact lens	Severe opacity; moderate edema; neovascularization extending to graft margin; epithelial defect 30–70% of graft; mostly dilated vessels; moderate irritation	Resolved/recovered	Possibly related	No
01-039	Male	62	Bacterial	6.5/6.5	ANCO-400-10	Transplant rejection; corneal melting	36 days	Medication treatment, wearing corneal contact lens	Moderate opacity; moderate edema; neovascularization around graft; epithelial defect <30% of graft; mostly dilated vessels; mild irritation	Resolved/recovered	Possibly related	No

^a^ The relationship between adverse events and “APCS graft” was categorized as “definitely related,” “probably related,” “possibly related,” “unlikely related,” and “unrelated.” Missing data were considered related adverse events. N/A = not applicable.

During the 6–12 months follow-up, two (3.4%) adverse events related to the trial occurred, including two cases of graft melting. One patient was treated with a conjunctival flap cover. The other patient underwent allogeneic corneal graft transplantation. With appropriate intervention, two out of the three cases fully recovered.

### Sensitivity analysis

After excluding five patients with visit window violations (subject No. 007, 008 from center 03 and subject No. 007, 011, 005 from center 04), the deblinding rate at 180 days post APCS transplantation was re-evaluated against the target value. The deblinding rate at 180 days was 91.0% [95% CI:81.5%, 96%], which was significantly higher than the target of 65% (*P* < 0.001) (Table 4).

**Table 4:** Sensitivity analysis of deblinding rate

Analysis set	Experimental group		
	Deblending rate 180 days after keratoplasty n (%)	95%CI	v.s. 0.65^a^
Per protocol set (n = 72)	65/72(90.3)	81%, 96%	<0.001
Modified ITT (Omit 5 patients lost to follow-up, n = 75)	61/67(91.0)	81.5%, 96.6%	<0.001
Modified ITT (Impute missing outcomes by last observation carried forward, n = 80)	62/71(87.3)	77.3%, 94%	<0.001

^a^ Fisher’s exact test was used to calculate the 95% CI and compare with the target value of 0.65. ITT = intent-to-treat population.

After imputing visual acuity data using the last observation carried forward method, the deblinding rate 180 days after APCS transplantation was evaluated against the target value. The results showed that the deblinding rate at 180 days was 87.3% [95% CI:77.3%, 94%], which was higher than the target value of 65% (Table 4). The results did not change substantially, confirming their robustness.

### One-way analysis

At 180 days after APCS transplantation, 65 patients (90.3%) achieved deblinding, while 7 patients (9.7%) remained blind. The mean BCVA (logMAR units) was 2.2 ± 0.3 in the deblinded group and 2.3 ± 0.3 in the blinded group, with no significant difference. Regarding corneal transplantation history, all 65 patients (100%) in the deblinded group had no prior corneal transplants, whereas 1 patient (14.3%) in the blinded group had undergone allogeneic corneal transplantation (*P* < 0.05). Subgroup analyses by infection type (bacterial, fungal, viral, and other), graft specifications (ANCO-250-10, ANCO-400-10, ANCO-500-10), and centers (01, 02, 03, 04) revealed deblinding rates exceeding the 65% target, except for the bacterial infection subgroup (50%, 1/2). No significant differences were observed in these factors (*P* > 0.05). Additionally, corneal opacity, ocular comorbidities, and allergy history did not show significant associations with outcomes (*P* > 0.05). Detailed results are provided in Table 5.

**Table 5:** One-way analysis (n = 72)

Characteristic		No. (%)	No. (%)	*P* value
	Deblinded at 180 days (%)	Deblinded at 180 days (n = 65)	Blinded at 180 days (n = 7)	
Primary corneal infection				0.2409
Bacterial (n = 2)	50	1 (1.5)	1 (14.3)	
Fungal (n = 51)	92.2	47 (72.3)	4 (57.1)	
Viral (n = 2)	100	2 (3.1)	0 (0)	
Other (undetermined pathogen) (n = 17) a	88.2	15 (23.1)	2 (28.6)	
Corneal clarity				0.2634
Grade 0 (transparent) (n = 0)	0	0 (0)	0 (0)	
Grade 1 (mild opacity) (n = 1)	100	1 (1.5)	0 (0)	
Grade 2 (moderate opacity) (n = 8)	100	8 (12.3)	0 (0)	
Grade 3 (severe opacification) (n = 63)	88.9	56 (86.2)	7 (100)	
Ocular comorbidities				0.974
None (n = 39)	89.7	35 (53.8)	4 (57.1)	
Cataract (n = 30)	90.0	27 (41.5)	3 (42.9)	
Glaucoma (n = 1)	100	1 (1.5)	0 (0)	
Strabismus (n = 1)	100	1 (1.5)	0 (0)	
Corneal dystrophy (n = 1)	100	1 (1.5)	0 (0)	
History of corneal transplantation				0.0022
None (n = 71)	91.5	65 (100)	6 (85.7)	
Yes (AALK) (n = 1)	0	0	1 (14.3)	
History of allergy				0.2886
No (n = 68)	91.2	62 (95.4)	6 (85.7)	
Yes (n = 4)	75	3 (4.6)	1 (14.3)	
Produce type				0.4256
ANCO-250-10 (n = 5)	100	5 (7.7)	0	
ANCO-400-10 (n = 59)	88.1	52 (80.0)	7 (100)	
ANCO-500-10 (n = 8)	100	8 (12.3)	0	
Study center				0.8898
01 study center (n = 38)	92.1	35 (53.8)	4 (57.1)	
02 study center (n = 6)	83.3	5 (7.7)	1 (14.3)	
03 study center (n = 11)	81.8	9 (13.8)	1 (14.3)	
04 study center (n = 17)	94.1	16 (24.6)	1 (14.3)	

AALK = allogeneic anterior lamellar keratoplasty.

^a^ Others refer to patients with no specific pathogens detected and unsuccessful empirical treatment for 2 weeks.

Variables are calculated by Pearson chi-square tests. *P* value <0.05 stands for statistically significant difference.

## Discussion

Through comparison of the deblinding rate at 180 days with the target rate of 65%, we found the post APCS transplantation value was 90.3%, which exceeded the target rate. The secondary outcomes investigated included vision, corneal transparency, and epithelial healing, all of which were found to be positive in the majority of cases, after a follow-up period of at least 6 months postoperatively. Furthermore, there were no clinically significant changes in laboratory parameters before and after treatment, indicating that APCS transplantation has sufficient efficacy and safety to meet clinical standards in the short to medium term post-transplantation.

### Appropriate clinical indication for APCS transplantation

APCS transplantation has been explored as an alternative to allogeneic corneal transplantation for the treatment of certain infectious corneal diseases. Initial reports by Zhang *et al* demonstrated the efficacy of APCS transplantation for fungal corneal ulcers and found risks of anterior chamber hypopyon and peripheral corneal ulcers preoperatively[8]. Since then, ophthalmologists have actively investigated appropriate indications for APCS transplantation. Chen *et al* compared the prognosis of central and peripheral corneal infectious ulcers using APCS transplantation, finding that patients with central ulcers experienced more rapid visual improvement, while patients with peripheral ulcers experienced higher incidences of immune rejection, graft melting, and neovascularization[16]. However, for small, peripheral corneal ulcers, APCS treatment was proven to be effective[17]. Subsequently, APCS transplantation was reported to be ineffective for treating non-infectious, peripheral corneal diseases like pterygium and Mooren’s ulcer, with persistent epithelial defects post-transplant and eventual graft melting[18]. At the same time, some studies have suggested that the high-risk factors for allogeneic corneal transplantation, active corneal infection[19] and a graft size greater than 9 mm[20], also increase the risk of APCS transplantation failure[21]. Large-area corneal transplantation indicates potentially more severe preoperative infection, and also increases the chance of immune reactions with the corneal limbus. In addition, APCS transplantation has also been attempted for advanced keratoconus but has shown inferior visual outcomes compared to allogeneic transplantation[22]. In summary, APCS transplantation is more suitable for central, mild to moderate infectious corneal disease^[23,24]^; however, for peripheral, extensive and severe infectious corneal disease, traditional allogeneic corneal transplantation may be preferable.

A significant advantage of this study over other studies is its more stringent selection of indications. Only patients with central mild-to-moderate infectious corneal disease without significant hypopyon were selected for APCS transplantation. Furthermore, unlike in previous studies which only used single-thickness APCS grafts[19], we utilized three thicknesses of products (graft sizes ranged from 3.5 to 9.0 mm) to match the depth of the corneal lesions. In contrast, the patients who received APCS grafts in previous studies had more complex conditions, including varying degrees of severe infection, hypopyon, and peripheral lesions, which may explain their higher postoperative complication rates^[8,16,18]^.

### Efficacy of APCS transplantation

Although decellularization processing is known to disrupt the regular arrangement of collagen fibers in porcine corneas[25], leading to reduced early transparency following APCS transplantation[12], chemical crosslinking technology forms stable chemical bonds between collagen fibers[26]. This not only strengthens APCS mechanically, with a tensile strength exceeding 1.00 MPa, but also alleviates postoperative edema, minimizes abnormal gaps between collagen fibers, and reduces moisture infiltration. Additionally, it helps restore the collagen fibers’ regular arrangement, enhancing optical transparency[27]. Compared to untreated APCS, crosslinked APCS demonstrated excellent transparency as early as 1 month post-surgery, with corneal transparency in most patients approaching that of allogeneic corneal transplant controls by 3 months[10]. Crosslinking technology significantly improves both the optical properties and structural stability of corneal grafts, supporting their clinical viability as a substitute for corneal transplantation^[8,12,28]^.

The treatment effects of APCS transplantation in patients with infectious corneal diseases include both visual acuity improvement and infection control. Previous studies reported an average logMAR BCVA of 1.18 at 12 months post-APCS transplantation[23] (Table 6). In comparison, allogeneic corneal transplantation for amebic corneal disease, assessed over an average of 5 months, yielded an average logMAR BCVA of 1.79[29] (Supplemental Digital Content, eTable 4, available at: http://links.lww.com/JS9/E723) Our study demonstrated that the average logMAR BCVA at 6 and 12 months after APCS transplantation were 1.10 and 1.17, respectively, showing superior outcomes compared to both allogeneic transplantation and APCS results from prior studies^[8,19,21]^.

**Table 6:** Summary of studies on APCS for corneal diseases

Author(s) and year	Research type	Study subjects	No. of eyes	Mean follow-up(mo)	Preoperative visual acuity (logMAR)	Postoperative best-corrected visual acuity (logMAR)	Recurrent infection and other complication
Chen Y *et al*, 2025	Retrospective case control study	Fungal keratitis	52	12	L2: 2.250 ± 0.303; L3: 2.381 ± 0.470	L2:1.18 ± 0.674; L3:2.11 ± 0.600	L2: Recurrence of infection 5%; Graft dissolution 15%; Graft rejection 0%; L3: Recurrence of infection 15.6%; Graft dissolution 28%; Graft rejection 9.3%
Yiming Hu *et al*, 2025	Prospective sequential study	Infectious keratitis	14	12	HM-0.2*	0.08-0.4* (3 months)	NR
Tian Liang *et al*, 2024	Retrospective sequential study	Peripheral bacterial /Fungal keratitis	18	12	0.99 ± 0.8	0.43 ± 0.3	Graft opacification 11%
Yingxin Chen *et al*, 2021	Retrospective sequential study	Infectious keratitis	45	12	2.36 ± 1.23	0.61 ± 0.21	Recurrence of infection 8.9%; Epithelial deficiency 31.1%; Graft dissolution 26.7%
Saiqun Li *et al*, 2020	Prospective sequential study	Infectious keratitis	27	12	1.23 ± 0.95	0.23 ± 0.18	NR
Qinxiang Zheng *et al*, 2020	Prospective sequential study	Fungal keratitis	25	12	2.16 ± 0.32	0.70 ± 0.51	Recurrence of infection 4%; Graft rejection 12%; Neovascularization 28%; Ocular irritation 4%
Saiqun Li *et al*, 2019	Prospective sequential study	Infectious keratitis	39	12	NR	NR	Recurrence of infection 12.8%; Graft failure 30.8%
Jiao Zheng *et al*, 2019	Prospective sequential study	Viral keratitis	13	15.1 ± 5.8	LP-0.02*	HM-0.5*	Neovascularization 84.6%; Graft dissolution 23.1%
Mingchang Zhang *et al*, 2015	Prospective single-arm cohort study	Fungal keratitis	47	6	NR	NR	Neovascularization 53%; Graft dissolution 8.5%; Ocular irritation 19%

L2 = 0.30 ± 0.05 mm APCS; L3 = 0.40 ± 0.05 mm; LP = light perception; HM = hand movements; NR = not reported.

^*^ Range of vision (decimal vision).

The recurrence rate of primary infection after allogeneic corneal transplantation ranges from 14% to 33% over 5–56 months,^[29–31]^ while it is 4–12.8% for previously studied APCSs at 12 months^[16,21,28]^ (Supplemental Digital Content, eTable 4, available at: http://links.lww.com/JS9/E723 and Table 6) Our study demonstrated that, with conventional corticosteroid and immunosuppressive treatments, primary infections were nearly fully controlled after APCS transplantation, outperforming allogeneic corneal transplantation. APCS transplantation promotes corneal repair and regeneration through its structure and biocompatibility, aiding the healing of infection-induced tissue damage. Additionally, precise indication selection, such as central, mild-to-moderate infectious corneal diseases, enhances therapeutic outcomes and reduces the risk of infection recurrence.

In addition to improving clinical outcomes, APCS offers significant cost-effectiveness, particularly in resource-limited settings[32]. Traditional human donor corneas are costly and in short supply, leading to extended waiting times and increased transplantation costs[33]. In contrast, APCS, derived from porcine corneas, can be produced in large quantities at lower cost and with a more reliable supply, making it a viable alternative in resource-constrained regions and reducing the economic burden of corneal transplantation[34]. Moreover, APCS’s superior mechanical strength and optical transparency may reduce postoperative complications and the need for re-transplantation, further enhancing its cost-effectiveness[26]. Therefore, APCS represents a cost-effective solution for treating infectious corneal diseases, delivering high-quality care while lowering the costs associated with traditional allogeneic transplantation.

### Safety of APCS transplantation

Safety factors of APCS transplantation include immune rejection and postoperative complication rates. The postoperative complication rate is related to the degree of decellularization[35]. Advantages of APCS are its inherently low antigenicity and immunogenicity, as cellular components and antigens are removed during decellularization[36]. Reported allograft rejection rates for infectious corneal disease ranged from 3.4% to 50% over 39–56 months^[30,31]^, compared to 12% for APCS transplantation at 12 months[28] (Supplemental Digital Content, eTable 4, available at: http://links.lww.com/JS9/E723 and Table 6) In our study, two (2.2%) patients had possible immune rejection related to APCS, with one ultimately requiring a second allograft. This rejection rate is significantly lower than those reported in previous studies^[21,28,31]^.

Previous studies have shown the most common APCS graft failures are due to primary infection recurrence, graft dissolution, and extensive neovascularization[21]. Graft dissolution is related to collagen degradation due to excessive release of tissue collagenases[37]. Compared with allogeneic corneas, decellularization of APCS exposes collagen fibers and makes them more susceptible to destruction by collagenases, leading to graft dissolution[38]. Previous APCS studies have reported epithelial defect and graft dissolution rates of 31.1% and 8.5–26.7% within 12 months^[8,16]^, with the epithelial defect rate comparable to that of allogeneic grafts, which range from 30% to 46.2% within 5–12.9 months^[29,39]^ (Supplemental Digital Content, eTable 4, available at: http://links.lww.com/JS9/E723) Our study reported the most comprehensive complication rates to date for APCS transplantation. The results showed that over 80% of patients achieved complete epithelial healing postoperatively, with low graft melting in only three (3.3%) patients. With treatment, two of the patients improved, with only focal epithelial defects at the final follow-up. In contrast to previous APCS studies reporting non-neovascularization rates of 16–72% within 12 months^[8,19,28]^ (Table 6), our study demonstrated a higher percentage, with 76.3% of patients free from corneal neovascularization at 6 months and 78.2% at 12 months. However, few long-term follow-up studies on APCS have been performed[40]. The final follow-up results also showed that the APCS grafts remained stable, with well-performed corneal epithelial healing and further improved transparency (Fig. 6), comparable to the surrounding normal corneas. Long-term follow-up revealed neural fiber ingrowth into the grafts (Fig. 6), which is consistent with previous study results[19].

### One-way analysis

This study included patients with infectious keratitis unresponsive to 2 weeks of antimicrobial therapy, with infections confined to the corneal stroma, representing a typical clinical indication for anterior lamellar keratoplasty. Thus, the cohort is highly representative of cases requiring surgical intervention, underscoring the applicability of APCS transplantation in such clinical settings.

Deblinding rates exceeded the predefined threshold of 65% across nearly all subgroups, including infection type (excluding bacterial), clinical center, and graft model. No significant differences were observed between deblinded and blinded patients, further supporting the efficacy of APCS transplantation. This may be attributed to standardized surgical protocols, which included uniform graft preparation, consistent suturing techniques, structured postoperative care, and comprehensive surgeon training[41].

At 180 days post-transplant, a history of prior corneal transplantation was the only factor significantly associated with deblinding. This finding aligns with prior literature, which suggests poorer outcomes in repeat transplants due to neovascularization and heightened immune responses[42]. Notably, cataracts, the most common comorbidity in this cohort, had minimal impact on outcomes, likely because they did not directly affect corneal graft function. However, the limited sample size and potential subgroup confounders warrant caution when interpreting these univariate analysis results.

### Limitations

This study has several limitations. First, the single-arm design precludes direct comparison with conventional treatments such as human donor corneal transplantation. While the observed 90.3% deblinding rate suggests promising efficacy, it may be influenced by patient selection bias or the lack of a control group. Future randomized controlled trials comparing APCS with other treatments are warranted to validate its comparative efficacy and long-term outcomes. Second, although our study included an extended 12-month follow-up for 75% of participants – during which high deblinding rates (92.9%) and favorable ocular surface outcomes were maintained – this duration may still be insufficient to fully assess late-onset complications such as immune rejection, progressive neovascularization, or stromal remodeling. These risks typically manifest over longer periods, and future studies with extended multi-year follow-up are warranted to further validate the long-term safety and durability of APCS transplantation. Third, changes in corneal thickness, endothelial cell loss, nerve regeneration, and improvements in quality of life and psychological well-being were not recorded or analyzed in detail. These metrics should be incorporated into future studies to provide a more in-depth evaluation. Finally, the trial was registered retrospectively due to initial oversight, reflecting the limited awareness of prospective registration in China at the time. While this may affect the ability to track protocol deviations, the study followed a predefined protocol and analysis plan, with complete outcome data for all participants. We acknowledge this limitation and emphasize our efforts to maintain transparency and methodological rigor throughout the trial.

## Conclusion

In conclusion, the APCS demonstrated high corneal transparency and nearly complete epithelialization post-keratoplasty, with the graft proving to be safe and effective. The six-month data indicate that APCS shows good efficacy and safety in the short to medium term, potentially offering a viable alternative treatment for patients with mild to moderate infectious corneal disease. However, further long-term randomized controlled trials are needed to confirm its non-inferiority and to assess chronic complications. These preliminary results provide strong evidence for continued clinical research.

## Data Availability

Data requests should be addressed by E-mail to the corresponding authors. The trial protocol will be made available.

## Research registration unique identifying number (UIN)

ClinicalTrials.gov identifier: ChiCTR2300075296 (registered 31 August 2023 – retrospectively registered; https://www.chictr.org.cn/.
